# Assessing the Impacts of Lead Corrosion Control on the Microbial Ecology and Abundance of Drinking-Water-Associated Pathogens in a Full-Scale Drinking Water Distribution System

**DOI:** 10.1021/acs.est.3c05272

**Published:** 2023-11-16

**Authors:** Isaiah Spencer-Williams, Mitchell Meyer, William DePas, Emily Elliott, Sarah-Jane Haig

**Affiliations:** † Department of Civil and Environmental Engineering, University of Pittsburgh, Pittsburgh, Pennsylvania 15261, United States; ‡ Department of Pediatrics, University of Pittsburgh School of Medicine, Pittsburgh, Pennsylvania 15261, United States; § Department of Geology and Environmental Science, University of Pittsburgh, Pittsburgh, Pennsylvania 15260, United States; ∥ Department of Environmental & Occupational Health, School of Public Health, University of Pittsburgh, Pittsburgh, Pennsylvania 15261, United States

**Keywords:** phosphate-based corrosion control, DWPI, opportunistic pathogen, drinking water, distribution system, *Legionella*, nontuberculous mycobacteria, drinking water microbiome

## Abstract

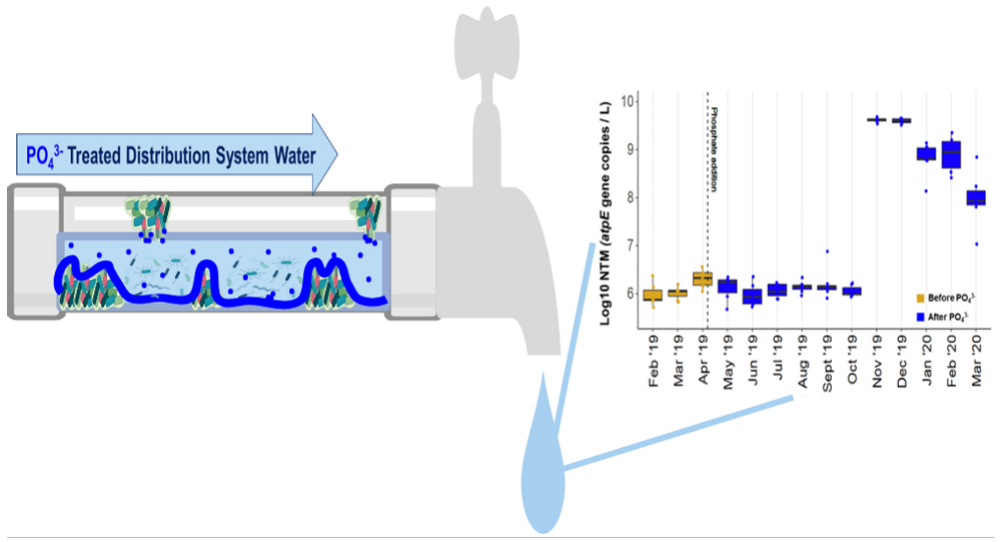

Increases in phosphate availability in drinking water distribution systems (DWDSs) from the use of phosphate-based corrosion control strategies may result in nutrient and microbial community composition shifts in the DWDS. This study assessed the year-long impacts of full-scale DWDS orthophosphate addition on both the microbial ecology and density of drinking-water-associated pathogens that infect the immunocompromised (DWPIs). Using 16S rRNA gene amplicon sequencing and droplet digital PCR, drinking water microbial community composition and DWPI density were examined. Microbial community composition analysis suggested significant compositional changes after the orthophosphate addition. Significant increases in total bacterial density were observed after orthophosphate addition, likely driven by a 2 log 10 increase in nontuberculous mycobacteria (NTM). Linear effect models confirmed the importance of phosphate addition with phosphorus concentration explaining 17% and 12% of the variance in NTM and *L. pneumophila* density, respectively. To elucidate the impact of phosphate on NTM aggregation, a comparison of planktonic and aggregate fractions of NTM cultures grown at varying phosphate concentrations was conducted. Aggregation assay results suggested that higher phosphate concentrations cause more disaggregation, and the interaction between phosphate and NTM is species specific. This work reveals new insight into the consequences of orthophosphate application on the DWDS microbiome and highlights the importance of proactively monitoring the DWDS for DWPIs.

## Abstract 2

Limited work has explored the impacts of full-scale distribution system orthophosphate lead corrosion control on drinking water microbiomes and pathogen abundance. This study reports changes in drinking water pathogen abundance and organism impacts after full-scale orthophosphate addition.

## Introduction

Lead contamination in drinking water supplies in the United States is a major concern, as millions of lead service lines remain in use.^1^ As such, many drinking water utilities have undertaken lead service line replacement alongside distribution system chemical corrosion control strategies (e.g., orthophosphate) to prevent further contamination and to remain in compliance with the Environmental Protection Agency’s Lead and Copper Rule.^2^ Typically, lead contamination is prevented via the formation of lead solids (e.g., PbO_2_) to reduce dissolved lead concentrations in the water; however, in the presence of disinfectants such as chlorine or chloramine, these solids may dissolve.^3^ Orthophosphate (PO_4_^3–^) is an established lead corrosion inhibitor^4^ that forms low-solubility lead solids (i.e., a protective scale). Alongside pH and alkalinity adjustments, PO_4_^3–^ is among the most widely used approaches for corrosion control due to its versatility in waters with variable water quality parameters, such as pH, alkalinity, and dissolved inorganic carbon.^3,5,6^ However, the addition of excess PO_4_^3–^ into the drinking water distribution system (DWDS, added in excess to promote scale formation, which can take weeks to months) may pose an unexpected challenge of increased microbial growth due to abundant nutrient availability.

DWDSs host a diverse aquatic ecosystem that can be altered by several factors including source water quality, temperature, treatment processes, disinfection methods, and piping materials.^7−9^ Furthermore, DWDSs are oligotrophic environments in which diverse microbial communities compete for limited nutrient availability. Thus, the introduction of excess bioavailable PO_4_^3–^ into DWDSs which are typically PO_4_^3–^ limited may aid microbial growth and cause changes in microbial community composition in the DWDS.^10,11^ For example, recent metagenomics studies in a United Kingdom DWDS found an increase in microorganisms that can more readily metabolize phosphate (e.g., *Candidatus accumulibacter*)^10,12,13^ after increased PO_4_^3–^ addition. Likewise, PO_4_^3–^ has been observed to be important for the growth of drinking-water-associated pathogens that cause infections in the immunocompromised (DWPI) such as *Legionella pneumophila*, *Pseudomonas aeruginosa*, and nontuberculous mycobacteria (NTM).^14−17^

DWPIs are typically defined as organisms that pose no threat to healthy individuals but can cause infection in immunocompromised people (e.g., those at opposite ends of the age spectrum and people with suppressed immune systems). DWPIs are not currently regulated by the U.S. EPA despite the fact they are a leading cause of morbidity and mortality in the U.S.^18^ whereby disease incidence exceeds that of traditionally monitored fecal-borne pathogens.^19−22^ DWPIs are often found in complex biofilm communities within the DWDS that can be affected by nutrient fluctuations and availability.^23,24^ As such, it is important to understand the potential impacts from the addition of nutrient-based corrosion inhibitors (i.e., PO_4_^3–^-based inhibitors) on both the DWPI density and biofilm formation (or aggregation) potential. From a public health standpoint, it is estimated that pulmonary infections associated with DWPIs annually cause >145000 infections^25^ and cost the economy >$62 billion in losses due to deaths alone based on a value of statistical life calculation using the Department of Health and Human Services central value.^26^

Despite the vast improvement in the understanding of the drinking water microbiome in the past decade,^27,28^ we still know relatively little about the impacts that large-scale changes in operation (e.g., the addition of PO_4_^3–^ corrosion inhibitors) have on the drinking water microbiome and microorganisms relevant to public health. As such, it is imperative that any operational changes in the DWDS also involve proactive DWPI monitoring. This study aimed to assess the impacts of PO_4_^3–^ corrosion control addition on the microbiome and DWPI density across multiple sites in a DWDS. It was hypothesized that the addition of excess PO_4_^3–^ into a phosphorus-limited DWDS would alter both the microbial community structure and density of DWPIs. Additional work was done to examine the impacts of PO_4_^3–^ corrosion control on NTM aggregation potential to observe potential impacts on biofilm formation.

## Methods

### Sample Information and Orthophosphate Addition Details

From February 2019 to March 2020, samples were collected from seven routine monitoring sites in a DWDS in Pittsburgh, PA, USA, all of which received water from the same drinking water treatment plant (Figure A1 in the Supporting Information). This plant treats surface water by using coagulation, sedimentation, filtration, and disinfection by chlorination. After disinfection, the treated water is pumped to a storage reservoir and then treated at a smaller treatment plant (microfiltration, UV light, and chlorination) before transport through the DWDS. The seven sites are in six different pressure districts representing residence times ranging from 59 to 229 h as estimated by a tracer study.^29^

Prior to April 2019, soda ash was used as the corrosion control agent in the DWDS. After a year-long model pipe loop study conducted with the Pennsylvania Department of Environmental Protection, the drinking water utility decided to switch their corrosion control over to PO_4_^3–^, as it was the more effective option given the water chemistry of the system. Prior to PO_4_^3–^ implementation, the utility conducted a 7 month DWDS flushing campaign beginning in September of 2018. PO_4_^3–^ was applied at three different locations throughout the DWDS: once directly after treatment in the treatment plant, once at a DWDS pump station, and once after treated water was distributed from one of the storage reservoirs. PO_4_^3–^ was applied into the DWDS in a step-down methodology over the course of 6 months, with a starting dosage of 3.0 mg/L PO_4_^3–^ in April 2019 to help ensure proper scale formation. As of September 2019, orthophosphate has been dosed at 1.8 mg/L PO_4_^3–^ for scale maintenance (Figure A2 in the Supporting Information).

### Sample Collection

1 L water samples from the seven distribution system monitoring sites were collected after flushing the faucet for at least 5 min and waiting for both the temperature and chlorine residual to stabilize.^30^ All samples were filtered within 1 h of collection through a 0.2 μm polycarbonate filter (Isopore Membrane Filters, EMD Millipore, Billerica, MA, USA), and the resulting filters were stored at −20 °C for DNA extraction. In reviewing the preliminary results, we observed a significant 2 log 10 increase in NTM density and determined that further testing would need to be performed to understand what caused this increase. As such, 12 L water samples were collected at the treatment plant and used for our NTM bench-scale reactor assays. Two separate sampling bottles (Nalgene, Waltham, MA) were filled with 6 L of water pre and 6 L post PO_4_^3–^ addition and stored on ice after collection and their chlorine residual was quenched using sodium thiosulfate before use.

### Water Quality

Fourteen water quality parameters were measured (Tables A1 and A2 in the Supporting Information) following standard methods.^31^ Free and total chlorine and PO_4_^3–^ concentrations at the tap were measured on site using a portable DR900 spectrophotometer (Hach, Loveland, CO, USA) (Figure A3 in the Supporting Information). Temperature and pH also were monitored on site using a portable pH and temperature meter (Hanna Instruments, Ann Arbor, MI, USA) (Figures A2 and A3 in the Supporting Information), and ATP was also measured on site using an AquaSnap Total ATP meter (Hygiena, California, USA). Total and dissolved concentrations of iron, manganese, copper, and lead were measured by inductively coupled plasma mass spectrometry (PerkinElmer NexION 300 ICP-MS, Waltham, MA). Prior to analysis, all dissolved metal samples were prepared by passing water through a 0.45 μm nylon syringe filter (Thermofisher, Waltham, MA) primed with 5 mL of sample. All analyses, except pH, temperature, and ATP were performed in triplicate.

### Microbial Analyses

#### Droplet Digital PCR (ddPCR)

DNA was extracted from the stored filters using the FastDNA Spin Kit (MP Biomedicals, Solon, OH) and stored at −20 °C until use. The density (number of gene copies per unit volume of sample) of total bacteria and Cyanobacteria was determined using digital droplet PCR (ddPCR) as previously described.^32^ Additional ddPCR assays for *L. pneumophila* (*Lmip* gene),^33^*P. aeruginosa* (*Orpl* gene),^34^ and NTM (*atpE* gene)^35^ were conducted using previously published primers (Table A4 in the Supporting Information).

ddPCR reactions were performed for all DNA samples (*n* = 98), alongside negative controls (DNA extraction, ddPCR, and filtration controls) and positive controls (gblocks of the target amplicons provided by Integrated DNA Technologies, Inc., Coralville, IA, USA), both of which were negative and positive, respectively (Table A3 in the Supporting Information). Positive samples were only determined above the LOD for each assay. 22 μL reactions contained 11 μL of 2× ddPCR Supermix (Bio-Rad Laboratories, Inc., Hercules, CA), 0.4 μM concentrations of all primers (Integrated DNA Technologies), 0.55 μL of bovine serum albumin (Invitrogen Corporation, Waltham, MA), and 2 μL of the DNA template. Droplets were generated to a 20 μL reaction volume using the automated droplet generation oil for Sybr (Bio-Rad Laboratories), and the plate was sealed. PCR was performed on a C1000 Touch thermal cycler (Bio-Rad Laboratories) within 15 min of droplet generation using the reaction conditions presented in Table A4 in the Supporting Information. Plates were run on a droplet reader within 1 h of PCR completion. Thresholds were set for each ddPCR assay (Table A3 in the Supporting Information) using Quantasoft v1.0.596 to determine the absolute density of the target taxa using the method described by Lievens et al.^36^

#### 16S rRNA Gene Amplicon Sequencing

16S rRNA gene amplicon (V4–V5 hypervariable region) library preparation and sequencing were performed on all samples (collected DWDS samples and negative controls) at Argonne National Laboratory following the Illumina Earth Microbiome Protocol.^37^ Samples were sequenced on an Illumina HiSeq2500 instrument with a total of 3301721 raw reads generated from the samples. Microbiome analysis was performed using QIIME2 with quality filtering performed using DADA2.^38^ Reads were assigned to operational taxonomic units (OTUs) using a 97% cutoff using the closed reference OTU-picking protocol in QIIME2 (version 2020.2) using the Silva (version 132.5) reference database. After OTU clustering, the presence of the OTUs in three or more of the negative control samples was removed (total of 97 removed) to ensure analysis only of the OTUs in the samples collected from the DWDS.

#### Bench-Scale NTM Growth Experiments

Eight 1.5 L glass batch reactors (Fisherbrand, Houston, TX) were set up to contain 1 L of drinking water obtained from the DWDS within 1 h of sample collection. Four reactors contained DS water pre PO_4_^3–^ injection, and the remaining four contained DS water post PO_4_^3–^ injection. All reactors were kept in dark conditions, placed on individual stir plates (Corning, Corning, NY) set to 300 rpm and run in parallel for 12 weeks until the reactors were out of water. To examine direct impacts on NTM growth, three different species of NTM that were found in the DS and have clinical or laboratory relevance were injected into the reactors: *Mycobacterium abscessus* (collected from a hospital ice machine, PA), *Mycobacterium avium* (collected from a monochloramine DWDS, MI^30^), and *Mycobacterium smegmatis* (strain mc^2^155,^39^ provided by the DePas lab, University of Pittsburgh). Each mycobacterial species was grown in liquid R2A media, with growth measured via optical density and plate counts prior to inoculation. Once grown, equal concentrations of all three liquid cultures were mixed, achieving a final concentration of 1 × 108 cfu/L (representative of the DWDS NTM average), which was injected into each reactor. Biweekly samples were collected from each reactor and processed for culturable NTM (evaluated by plate counts on Middlebrook 7H11 media plates, following standard procedures) and total NTM, *M. smegmatis*, *M. abscessus*, and *M. avium* absolute density by ddPCR using previously published primers (Table A4 in the Supporting Information).

#### NTM Aggregation Assays

To determine the impacts of PO_4_^3–^ addition on NTM aggregation (biofilm formation), an assay developed by DePas et al.^40^ was utilized to distinguish and quantify aggregated cells and planktonic cells over time. Briefly, NTM cultures were grown in tryptone-yeast extract magnesium sulfate (TYEM) nutrient broth prior to starting the experiment to ensure the same starting concentration. Once grown, liquid culture replicates (*n* = 3 in each experiment, 3 total experiments for *n* = 9 for each NTM species) were grown in fresh TYEM nutrient broth for 35 h (as previous work^40^ showed peak NTM dispersal within 35 h) with different concentrations of PO_4_^3–^ (0, 1, 20, and 100 uM). Cultures were then harvested by passing the culture through a 10 μm (*M. smegmatis*) or 5 μm cell strainer (*M. abcessus*, *M. avium*), and the optical density (OD_600_) of both the planktonic fraction (i.e., cells that passed through the strainer) and the aggregates collected on the strainer were recorded. The OD_600_ value of the planktonic fraction was immediately recorded, while aggregates that collected on the strainer were resuspended in phosphate-buffered saline (PBS) with 6% Tween20 (Sigma-Aldrich, St. Louis, MO, USA). This suspension was then sonicated to resuspend remaining aggregates before recording the OD_600_ value . Both OD_600_ readings were used to calculate the planktonic to aggregate ratios. Average planktonic/aggregate ratios were then compared across phosphate concentrations using nonparametric Wilcoxon testing (significance denoted at *p*-value <0.05).

### Statistical Analyses

Taxonomic and OTU tables generated for the samples were transformed using the Hellinger transformation due to the data set having many rare OTUs (present in a few samples) or low-abundance OTUs (i.e., less than 10% relative abundances^41^). The transformed OTU data were then used to calculate pairwise dissimilarities between samples based on the Bray–Curtis dissimilarity index. Resulting matrices were examined for temporal and spatial patterns in the bacterial community structure by Nonmetric Multidimensional Scaling as implemented in the Vegan package (version 2.5-7) in R (version 4.0.2).^42^ Significant differences in the microbial community compositions (Hellinger transformed OTUs) before and after PO_4_^3–^ addition were determined by nonparametric permutational multivariate analysis of variance (PERMANOVA) and differential abundance analysis using DESeq2.^43^ Relationships between environmental parameters and patterns in microbial community composition were examined by canonical correspondence analysis (CCA) with significance tested by ANOVA after removing collinear variables (variance inflation factor analysis value <10) and reducing the overall suite of environmental variables with a stepwise Akaike information criterion model. Additionally, significant differences in the relative and absolute bacterial density before and after PO_4_^3–^ addition and differences in NTM species aggregation were determined by nonparametric Wilcoxon testing, while the functional relationships between water quality parameters and bacterial groups were analyzed by stepwise multivariate forward/reverse regression analysis. All statistical analyses were performed in R (version 4.0.2)^44^ with significance set at a *p*-value <0.05.

## Results and Discussion

### Impacts of PO_4_^3–^ Addition on DS Microbial Community Composition

**Figure 1:**
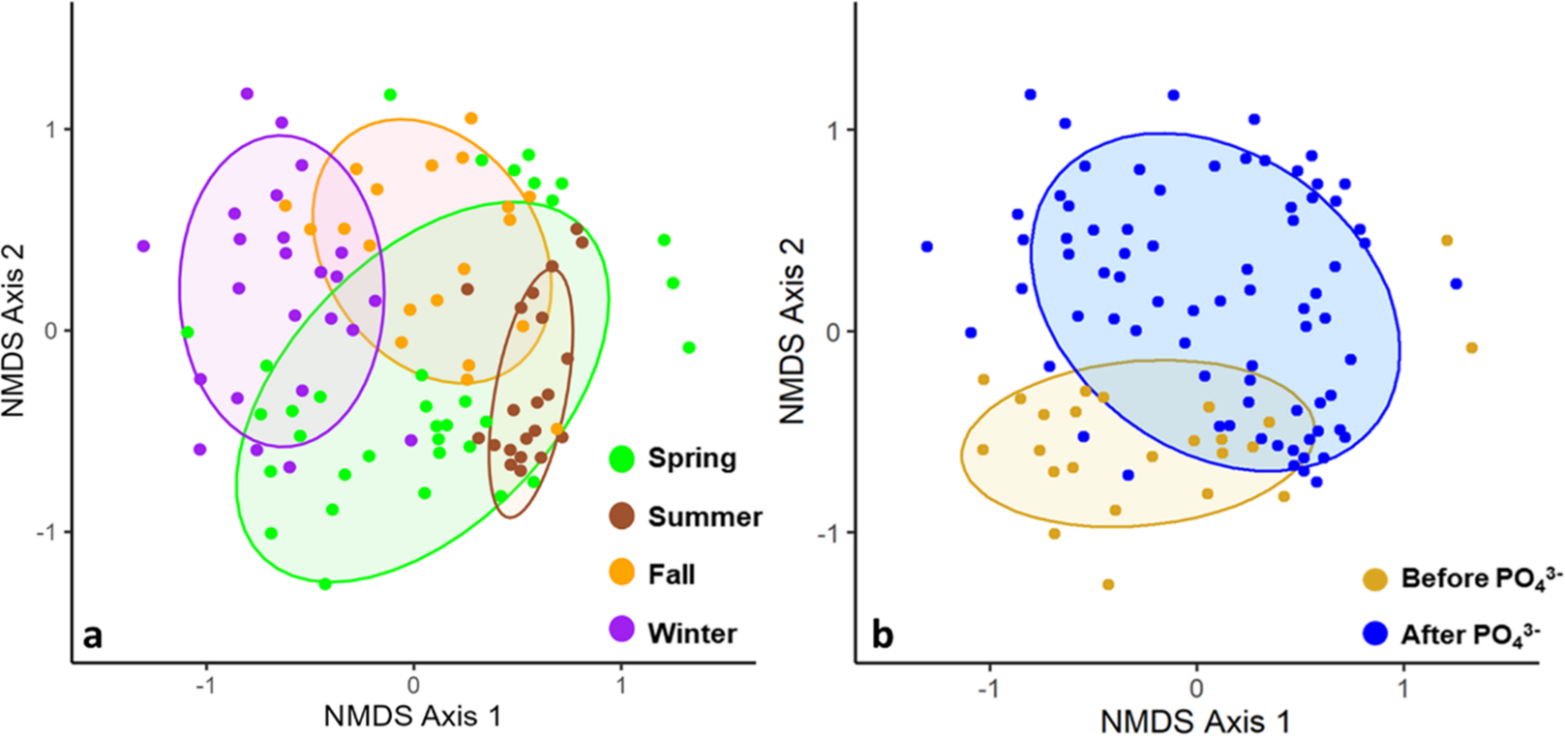
Nonmetric multidimensional scaling plots for all DS samples separated by (a) season and (b) orthophosphate addition into the DS (stress values = 0.26). The ellipses represent the 95% confidence interval of the distribution from the centroid of the cluster of points. Significant temporal and treatment variations were observed in the DWDS microbial community structures.

For the 98 samples collected from the DWDS over the course of 1 year, NMDS (Figure 1a,b) and PERMANOVA analysis on the 16S rRNA gene amplicon sequencing data showed significant seasonal (*r*^2^ = 0.08, *p*-value <0.001) and pre and post PO_4_^3–^ addition (*r*^2^ = 0.035, *p*-value <0.001) variation in the DWDS microbial community structures. The observed seasonal differences in microbial community composition in the DS are consistent with previous studies,^9,45^ and the observed differences in communities based on PO_4_^3–^ dosing corresponds with results found by Douterlo et al.^10^ Additionally, significant spatial differences were observed (*r*^2^ = 0.07, *p*-value <0.001), which is to be expected as each DWDS site has a different residence time and differing hydraulics and plumbing materials; however, these spatial differences were only driven by temporary differences at one or two sites.

**Figure 2:**
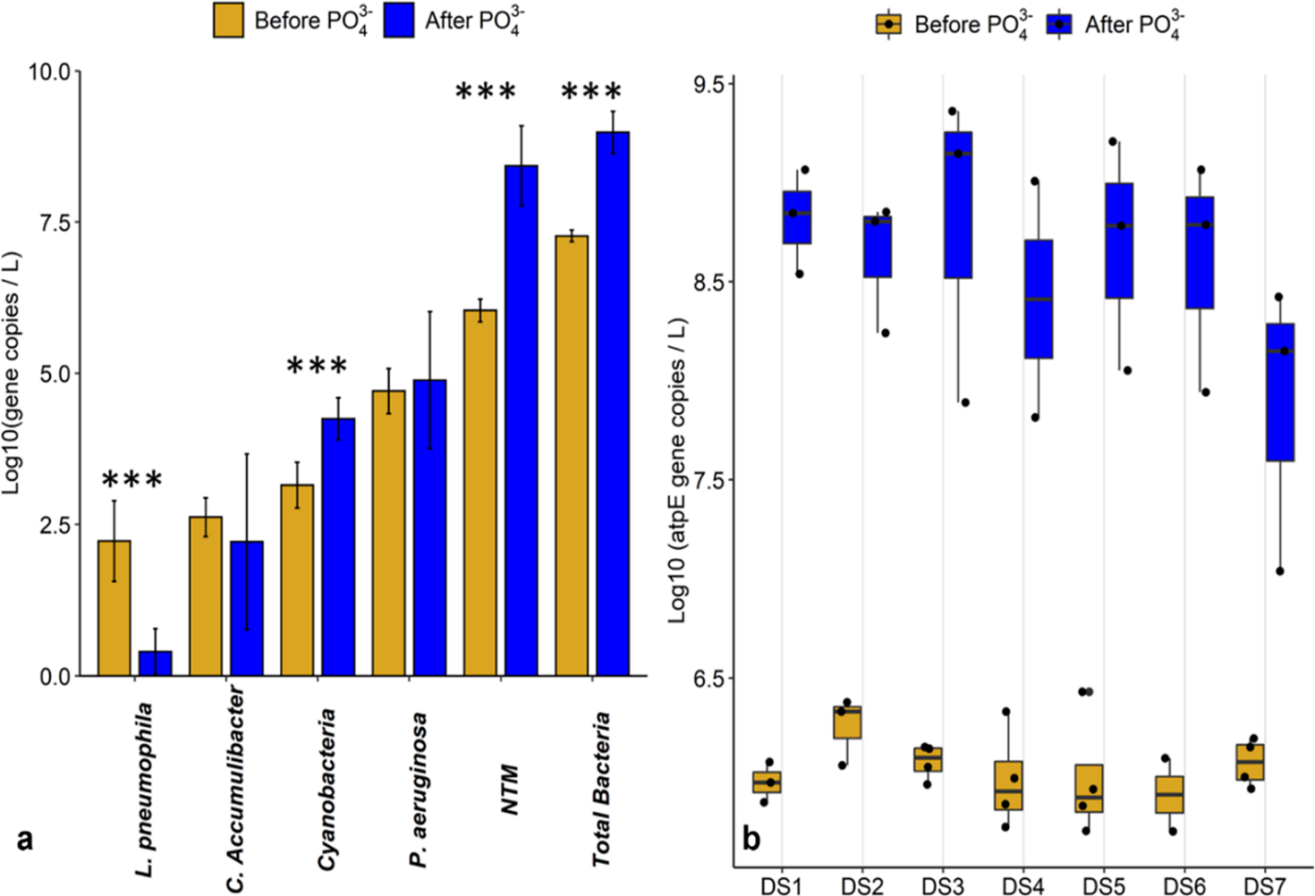
(a) Geometric average (*n* = 14) of absolute density of DWPIs, total bacteria, Cyanobacteria, and *C. Accumulibacter* in the DWDS before and 1 year after PO_4_^3–^ addition. Error bars represent the standard deviation. *** signifies a significant difference in measured density at *p-*value <0.001. (b) Box plot of NTM absolute density at each DWDS site before and 1 year after PO_4_^3–^ addition. In both graphs, the pairwise data (i.e., February and March 2019 before and February and March 2020 1 year after PO_4_^3–^ addition) was used to control for seasonal fluctuations in density.

The average relative abundance of the most abundant taxa in the DWDS was consistent with other studies,^46−48^ with Proteobacteria and Actinobacteria dominating across all DWDS sites. Furthermore, the *Acinetobacter*, *Pseudomonas*, and *Sphingomonas* genera were the only three genera within the top 10 most abundant taxa present both before and after PO_4_^3–^ addition (SI Figure A4). Apart from Cyanobacteria, differential abundance analysis revealed no other significant changes in the relative abundance of typical drinking water phyla. At the genus level, there were multiple significant changes in the abundance of rare drinking water taxa (i.e., *Nevskia*, *Stenotrophomonas*) and other uncultured organisms that could not be identified further. Cyanobacteria (particularly nonphotosynthetic relatives such as Melainabacteria, which made up a third of the Cyanobacteria present) generally represented 10% or less of the DS microbial community and appeared to significantly decrease (9% before PO_4_^3–^ addition, 2% after PO_4_^3–^ addition, *p-*value = 0.02) after PO_4_^3–^ addition into the DWDS. However, while a decrease in Cyanobacteria relative abundance was observed, there was also an unexpected significant increase in Cyanobacteria absolute density (Figure 2). The discrepancy between the two assays (16S rRNA amplicon sequencing and ddPCR) could be due to the selected primers amplifying organisms not classified as Cyanobacteria by the SILVA database. The observed significant decrease in Cyanobacteria relative abundance is, however, consistent with results from nutrient limitation assays conducted using water from the DWDS by Balangoda et al.^49^ Specifically, it was observed that Cyanobacteria grown in PO_4_^3–^-treated DWDS waters collected 1 year after PO_4_^3–^ addition only increased in growth when provided with additional nitrogen treatment. This observation suggests that nutrient limitations shifted for Cyanobacteria in the DWDS, going from nitrogen–phosphorus colimitation to strict nitrogen limitation. In other studies^50,51^ conducted in lake systems, similar observations of inhibited cyanobacterial growth in the presence of specific elevated nutrients have been observed. As such, the decrease in the relative abundance of Cyanobacteria in the DWDS could suggest a shift in the nutrient limitation in the DWDS, although further work is warranted to understand, evaluate, and better maintain nutrient limitations in the DWDS to control microbial presence and to better evaluate impacts on Cyanobacteria and related organisms abundance in drinking water such as Melainiabacteria like *Vampirovibrio* spp.,. Additionally, further work is warranted to better identify Cyanobacteria (both photosynthetic and nonphotosynthetic species) and related species in complex environmental matrices.

The few significant changes in typical drinking water taxa observed in the DWDS microbial relative abundance in this study could be a result of the short duration of the study, as 1 year may not have been enough time to see any drastic impacts in microorganism abundance. Furthermore, although all DWDS monitoring sites receive water from the same treatment plant, the residence time varies widely between them, which could also have an impact on the types of organisms present.^52^ The response in only a few typical taxa and many more rare (i.e., less abundant or abundant at small amounts) could also be indicative of shifts in microbial niches and function in response to elevated phosphate concentrations (and in turn, changes in nutrient limitations, C:N:P ratios, etc.) rather than shifts in taxonomic composition. CCA revealed that 16.4% of the variance in the microbial community composition could be explained by a combination of factors, including the geographic location of the DS sites, the season samples were collected in, pH, total copper concentration, total iron concentration, and total phosphorus (Table 1). Spatiotemporal (i.e., season and site location) variation was expected, as previous work has highlighted the impacts of spatial,^8,9,45^ temporal,^8,53^ and seasonal effects^9,54^ on the drinking water microbial community processes. Likewise, the impacts of pH, phosphorus, and dissolved metals have been shown to impact the DS microbiome.^10,12,13,15,23,45^

**Table 1:** Linear Effect Models for the Whole Distribution System Community Composition and Absolute Density of NTM and *L. pneumophila* Using All the Distribution System Water Samples Collected (*n* = 98) over the Course of 1 Year

taxa	data transformation^a^	model components^b^	explained by model (%)^c^
community composition (OTUs)	Hellinger(*x*)	DWDS site location^6.38%^ ± season^3.57%^ – pH^1.41%^ + total copper^1.32%^ + total iron^1.25%^ – total phosphorus^2.46%^	16
NTM	*x*^–0.3^	season^29%^ + total phosphorus^17%^ – *L. pneumophila*^16%^ – pH^6%^ – total iron^3%^ + turbidity^3%^	74
*L. pneumophila*	*x*^0.2^	season^36%^ – NTM^16%^ – total phosphorus^12%^ + total iron^4%^ – total chlorine^4%^ + pH^3%^ – turbidity^1%^	76

a NTM and *L. pneumophila* concentrations were transformed using the Box-Cox method in R to ensure normal distributions of the data for the models.

b Superscript numbers proceeding each component in the models show their relative percent contribution to the overall model.

c Percentage explained pertains to the adjusted *R*^2^ for the overall model. All models were significant at *p*-values <0.001.

### Impacts of PO_4_^3–^ on Bacterial Density and DWPI Density

As expected, the absolute density of total bacteria significantly increased 1 year after PO_4_^3–^ addition into the DWDS with a 50-fold increase in observed density (Figure 2a and Figure A5 in the Supporting Information). This change was likely driven by a 2 log 10 increase in the NTM density at all DWDS sites (Figure 2b). Interestingly, during this same time frame a significant decrease in *L. pneumophila* density was observed across all DS sites (Figure 2a), likely due to a significant decrease in the frequency of detection (100% detected before PO_4_^3–^, 62% detected after PO_4_^3–^, *p*-value <0.001). Previous work has discussed the negative correlation between *Legionella* spp. and *Mycobacterium* spp.^54^ that may be due to both DWPIs competing for the same nutrients in oligotrophic environments. Further regression analysis on *L. pneumophila* density revealed 76% of the variance in *L. pneumophila* was explained by a combination of factors including NTM density (Table 1), further suggesting their proposed antagonistic relationship.

Interestingly, sequencing results (relative abundance) of both *Actinobacteria* (3% decrease, *p-*value = 0.073) and *Mycobacterium* (1.5% increase, *p-*value = 0.483) do not reflect this significant increase, suggesting that community changes were driven at the subgenus level, as reflected by ddPCR results. It is important to also note that since our sequencing assays targeted the 16S rRNA gene which can vary between organisms, another possibility is that *Actinobacteria* and *Mycobacterium* differences may be masked by biasing toward changes in organisms with higher 16S rRNA copies (*Mycobacterium* have 1 copy of the 16S rRNA gene compared to the average 5.3 copies in bacteria in general). Possibly with metagenomics, we may have observed changes in the *Mycobacterium* genus, and future studies should take this into consideration when designing and planning to analyze these organisms. It is also important to note that the differences in assay target can make it challenging to analyze and compare these organisms. When doing amplicon sequencing (typically 16S rRNA), the data are generally reported as the relative abundance of the 16S rRNA gene copies present, while in ddPCR assays your gene target can be more specific (i.e., the *atpE* gene, the *hsp65* gene). This could also account for differences in assay results and should be considered when evaluating changes in taxa at lower order taxonomic levels (e.g., genus, species).

Previous work has detailed the ability of *Actinobacteria* species to solubilize and uptake phosphorus in soil, freshwater, and marine environments,^55−57^ and as such the observed significant increase in NTM density could result from the freshwater origins of the drinking water. Additionally, other work has shown a positive correlation between *Actinobacteria* abundance in drinking water systems and total phosphorus concentration^58^ while previous metagenomic work has suggested that *Actinobacteria* may have a key role in phosphorus sequestration^59^ in the environment. Regression analysis on the NTM density data revealed that 75% of the variance in NTM density was explained by a combination of seasonality, total phosphorus concentration, *L. pneumophila* density, pH, total iron concentration, and turbidity (Table 1). Previous work has highlighted that NTM are impacted by factors such as season,^60^ turbidity,^61^ pH, nutrient availability, and metal concentrations.^62−64^ Interestingly, however, the observed increase in the NTM (Figure A6 in the Supporting Information) also coincided with a decrease in the water temperature (Figure A2 in the Supporting Information). This observation was counterintuitive, as previous NTM work has reported seasonal increases in NTM during warmer times of the year^60^ and positive correlations with warmer water temperatures.^65^ As such, elucidating the reason for the observed increase in the NTM was important.

### Impacts of PO_4_^3–^ on NTM Growth and Aggregation Potential

Since the 2 log 10 increase in NTM density was observed suddenly across all DWDS sites starting in November 2019 (Figures A6 and A7 in the Supporting Information), further bench-scale experimentation on NTM growth and aggregation (biofilm formation) potential in the presence and absence of PO_4_^3–^ was conducted to elicit the impacts of PO_4_^3–^ on NTM. Both viability (plate counts) and ddPCR analyses revealed no significant differences in total NTM or NTM species (*M. smegmatis*, *M. avium*, and *M. abscessus*) density between samples treated with or without PO_4_^3–^ (Figure A8 in the Supporting Information). Interestingly, *M. smegmatis* was detected in only 9% of the samples taken, possibly because the laboratory mc^2^155 strain was outcompeted by the environmental *M. abcessus* and *M. avium* strains and other DW microbiota. Given the sudden increase in the total NTM density in the DWDS, the lack of significant differences in batch reactor NTM growth and decrease in NTM density 3 years later (Figures A6 and A7 in the Supporting Information), it was unlikely that the PO_4_^3–^ addition caused a significant impact on NTM growth. Instead, it is possible that the PO_4_^3–^ addition impacted the NTM biofilm processes (formation and sloughing).

Biofilm formation is a complex, dynamic, and ongoing process that has several stages, some of which are dependent on nutrient concentrations.^66,67^ As nutrient concentrations shift, it is possible for organisms within biofilms to disperse into the planktonic phase and recolonize in areas where conditions are more suited to biofilm growth.^67^ Additionally, previous work has suggested that a lack of phosphate triggers the expression of genes and metabolic pathways relating to mycobacterial cell aggregation,^68^ while a clinical study mentioned use of PBS to keep their mycobacterial cultures in suspension.^69^ Given these considerations, it was hypothesized that increasing phosphate concentrations could impact the aggregation potential of NTM species, and the stepdown in PO_4_^3–^ dose in the DWDS (starting dose = 3.0 mg/L, scale maintenance dose = 1.8 mg/L) could likely be an explanation for the observed increase.

**Figure 3:**
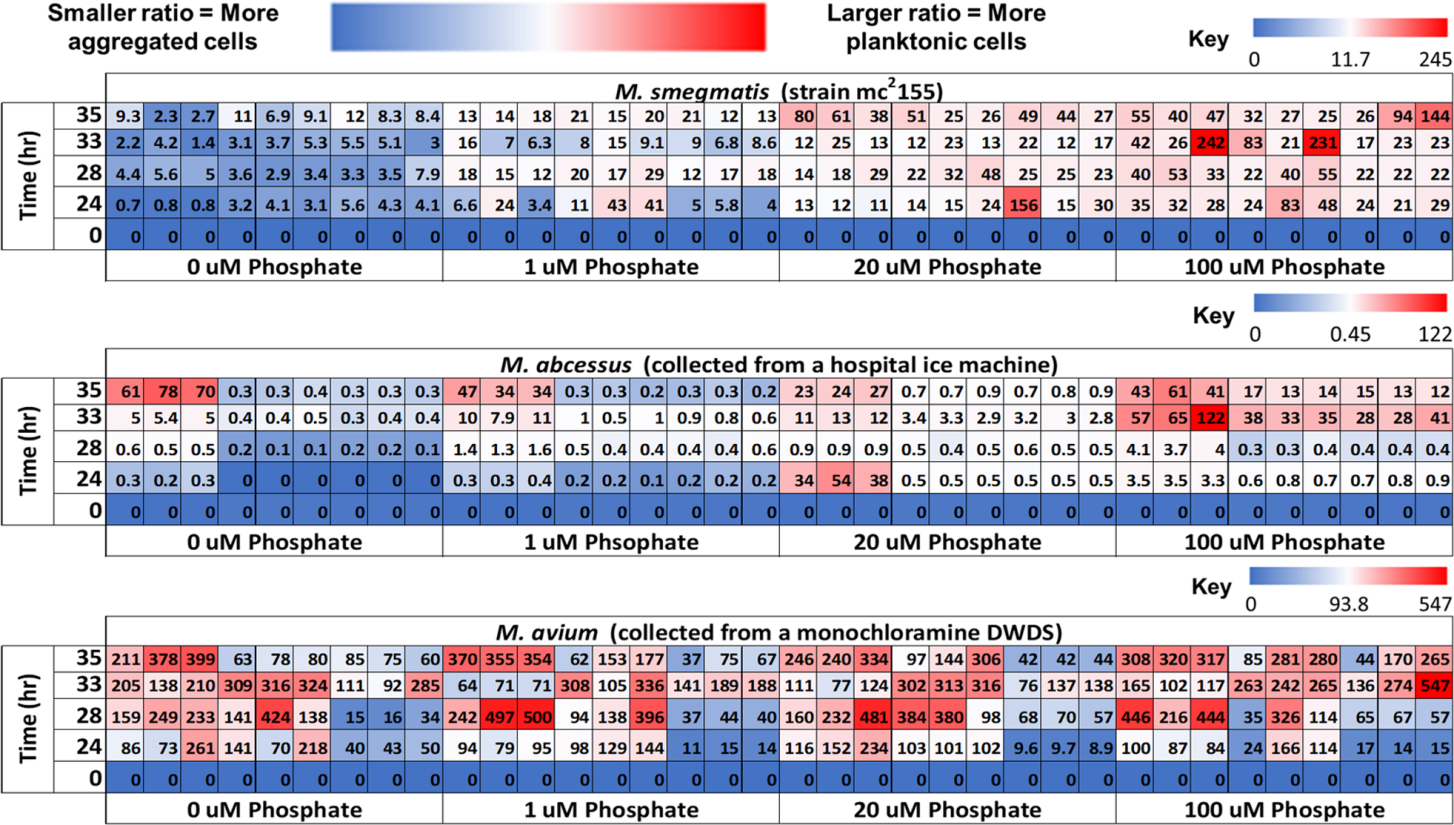
Planktonic vs aggregate NTM ratios (ranges in parentheses) for *M. smegmatis* (0–245), *M. abcessus* (0–122), and *M. avium* (0–547) at different concentrations of phosphate (each tile is one technical replicate, for a total of *n* = 9 per species × phosphate concentration × time). Blue cells represent a smaller ratio, signifying a larger proportion of aggregated NTM cells, while white/red cells represent a higher ratio, signifying a larger proportion of planktonic NTM cells. The white cells represent the 50th percentile of each specific species data set. The ratios were obtained by dividing the planktonic OD_600_ measurements by the aggregate OD_600_ measurements.

To elucidate the impact of phosphate on NTM aggregation, a comparison of planktonic and aggregate fractions of NTM cultures grown at varying phosphate concentrations was conducted following the protocol developed by Depas et al. Briefly, this involved growing NTM cultures in TYEM broth augmented with varying phosphate concentrations and passing the culture through a cell strainer to assess aggregation over the incubation time. The OD_600_ values of the planktonic NTM (NTM that passed through the strainer) and aggregated NTM (NTM trapped on the strainer) fractions were measured and compared by assessing the ratio of the NTM in the planktonic phase to the NTM aggregated phase (hereafter referred to as “NTM aggregation assays”). In nutrient-rich media (primarily C:N dominated), previous work with domesticated *M. smegmatis* has observed that NTM initially aggregates but will then disperse at later culture maturity and grow planktonically.^40^ In the presence of phosphate-augmented media, the domesticated *M. smegmatis* behaved similarly to what was observed by DePas et al.; however, *M. abcessus* and *M. avium* behaved differently (Figure 3).

The impacts of different phosphate concentrations on NTM species dispersal seem to be species dependent, as the results of the NTM aggregation assay on *M. abcessus* revealed an increased ratio with higher phosphate concentrations, while the same trend was not observed with *M. avium*. The NTM aggregation assays for *M. abcessus* revealed significantly (*p*-value <0.001) lower average ratios (more aggregate fractions) in the 0 and 1 μM phosphate conditions compared to the 20 and 100 μM conditions ( and Figures A9 and A10 in the Supporting Information). The increased ratio in the presence of elevated phosphate suggests that increased phosphate concentrations may cause disaggregation (potentially biofilm sloughing) for *M. abcessus*. In the *M. avium* assays, elevated ratios were present regardless of the phosphate concentration (Figure 3), suggesting that phosphate concentration may not have as much of an impact on *M. avium* aggregation.

The differences observed in NTM species could be attributed to differences in nutrient requirements between rapid- and slow-growing NTM, as one study posits that slow-growing mycobacteria (e.g., *M. avium*) may not benefit from elevated phosphorus concentrations when compared with rapidly growing mycobacteria.^68^ The results of the aggregation assay data presented here are interesting, as previous work has mentioned the impact of phosphate concentration on the production of extracellular polymeric substance (EPS)^12,70^ and biofilm structural mechanics.^71,72^ Specifically, it has been reported that increasing phosphorus concentrations can result in a reduction in EPS or an increase in the amount of pores present in the biofilm, thus weakening biofilms and making them more susceptible to detachment under drinking water pipe flows.^71^ Additionally, previous spatial work has demonstrated that NTM are late colonizers of biofilms in drinking water.^73^ Therefore, it is possible that NTM could be on the outer surface of drinking water biofilms, making detachment easier; however, more work is needed on the localization of NTM in drinking water biofilms. Furthermore, it is also possible that phosphate interacts with the constituents of the outer membrane of mycobacterial cells and causes a change in the cell hydrophobicity; however, to the authors’ knowledge this has not been explored.

Given the data presented, it is possible that the sudden increase in NTM in the DWDS was due to a large biofilm sloughing event, an interaction between phosphate and the NTM cell walls, or a combination of both. Moreover, given what appears to be a gradual decrease to a new increased baseline NTM density (Figure A6 in the Supporting Information), it is also important to consider the time scales of impact. Previous work has examined the impact of nutrient starvation on biofilm formation and suggested that prolonged nutrient starvation can lead to increased biofilm detachment.^74^ In the presented system, the shift from excess PO_4_^3–^ dosed at 3.0 mg/L down to 1.8 mg/L could have been enough of a shift to trigger a starvation response from the biofilm. It could also be possible that the length of the elevated NTM concentrations was a delayed response to the change in DWDS PO_4_^3–^ concentration (Figures A6 and A7 in the Supporting Information). Future studies should identify the drivers of NTM disaggregation in the presence of elevated phosphate concentrations, create a more normalized process for examining disaggregation that captures robust species dynamics, and elucidate the species differences in biofilm formation potential, as well as determine the time scale of sloughing events in the DWDS.

Overall, the results presented here suggest that PO_4_^3–^ addition into the DWDS temporarily increased the total number of bacteria present in the DWDS and altered community structure with respect to an increase in NTM and decrease in *L. pneumophila* density. The findings presented provide an interesting basis for the continued monitoring of DWPIs in nutrient-limited water treated with PO_4_^3–^ corrosion control and also demonstrate the need for surveillance during operational changes. Furthermore, as PO_4_^3–^ addition has been shown to elicit a 2 log 10 increase in NTM density in a full-scale DS, likely driven by biofilm disaggregation from the results of the aggregation assays, more work is needed to understand the mechanisms driving this process. Future studies should consider using enhanced setups (e.g., pipe or continuous flow reactors with DWDS materials) to (1) conduct more targeted analysis (ddPCR and sequencing of different genes, e.g., hsp65), (2) determine what specific types of NTM or other DWPIs are present in a full-scale DWDS, and (3) continue to develop our understanding of the impacts of nutrients on these organisms and biofilm formation. Likewise, although no changes in NTM pulmonary disease incidence have been observed to date, additional longitudinal studies are required to ensure that no adverse health impacts arise.

## Data Availability

The environmental and sequencing data that support the findings of this study are openly available in Zenodo at 10.5281/zenodo.8111150, under reference number 8111150.

## Supporting Information Available

Additional information, tables, and figures including water quality data and ddPCR information (PDF)
