# Supplementary material for: Assessing the Impacts of Lead Corrosion Control on the Microbial Ecology and Abundance of Drinking-Water-Associated Pathogens in a Full-Scale Drinking Water Distribution System
Source: Environ Sci Technol. 2023 Nov 16;57(48):20360–9. doi: 10.1021/acs.est.3c05272 (PMC10702490; doi:10.1021/acs.est.3c05272)
Supplement: Supplementary file 1 — es3c05272_si_001.pdf [file es3c05272_si_001.pdf]

Supplementary Information

Assessing the Impacts of Lead Corrosion Control on the Microbial Ecology and Abundance of Drinking Water Associated Pathogens in a Full-Scale Drinking Water Distribution system

Isaiah Spencer-Williams[1], Mitchell Meyer[2], William DePas[2], Emily Elliott[3], Sarah-Jane Haig[1,4,]*

[*]*Corresponding author, sjhaig@pitt.edu*

[1]*Department of Civil and Environmental Engineering, University of Pittsburgh, Pittsburgh, PA, 15261*

[2]*Department of Pediatrics, University of Pittsburgh School of Medicine, Pittsburgh, PA, 15261*

[3]*Department of Geology and Environmental Science, University of Pittsburgh, Pittsburgh, PA, 15260*

[4]*Department of Environmental & Occupational Health, School of Public Health, University of Pittsburgh, PA, 15261*

Summary of contents: 15 pages, 10 Figures, 5 Tables

TABLE OF CONTENTS

Figure A1: Map of distribution system routine monitoring site sampling locations.

Figure A2: PO_4^{3-} dosing (left scale, in blue) and water temperature (right scale, in orange) over the duration of the one-year study. Water temperature was averaged between the seven distribution sites and error bars represent the standard deviation.

Figure A3: Total chlorine (a), Total lead (b), pH (c), and PO_4^{3-} concentration (d) across the seven distribution system sites over the duration of the one-year study.

Figure A4: a) Top 10 most abundant phyla and **b)** top 10 most abundant genera across all DWDS sites before and 1-year after full-scale PO_4^{3-} addition (n = 14 in each condition). To control for seasonality, only the matching months before and after PO_4^{3-} application were compared.

Figure A5: Average ± standard deviation of absolute density of DWPIs, total bacteria, and Cyanobacteria in the DWDS before (n = 21) and after PO_4^{3-} addition (n = 77). The after category contains data from 9 months of sample collection.

Figure A6: Absolute NTM (a) and Total bacterial (b) density in the DWDS throughout the study duration and comparison three years later. *Note: only six samples were collected in 2022, as one of the routine monitoring sites has been shut down*

Figure A7: Ratio of NTM to total bacteria across the seven distribution system sites over the course of the study duration and three years later. *Note: only six samples were collected in 2022, as one of the routine monitoring sites has been shut down*

Supplementary Information

Figure A8: a) NTM concentration over the study duration and **b)** absolute density of NTM species in batch reactors. Over the duration of the experiment (~ 3 months), a significant difference in *M. smegmatis* density was observed, likely due to competition with the environmental *M. avium, M. abcessus,* and other microorganisms present in the water.

Figure A9: Aggregate fraction of *M. abcessus* cultures suspended in phosphate buffered saline mixed with 6% Tween20. As phosphate level increased, the number of suspended aggregates decreased.

Figure A10: Average (n = *9 for each species, at each timepoint, at each phosphorus concentration*) ± standard deviation of planktonic vs aggregate NTM ratios for *M. abcessus* (left), *M. avium* (middle), and *M. smegmatis* (right). Ratios were obtained by dividing the planktonic OD600 measurements by the aggregate OD600 measurements. A larger ratio signifies a larger amount of NTM in the planktonic phase.

Table A1: Different water quality parameters measured in this study and the method / apparatus used

Table A2: Average ± standard deviation measured water quality parameters before and after full-scale PO_4^{3-} addition into the DWDS.

Table A3: ddPCR target genes, amplicon size, annealing temperature, primer sequences, and references

Table A4: ddPCR assay thresholds, LOD, and LOQ

Table A5: ddPCR reaction conditions

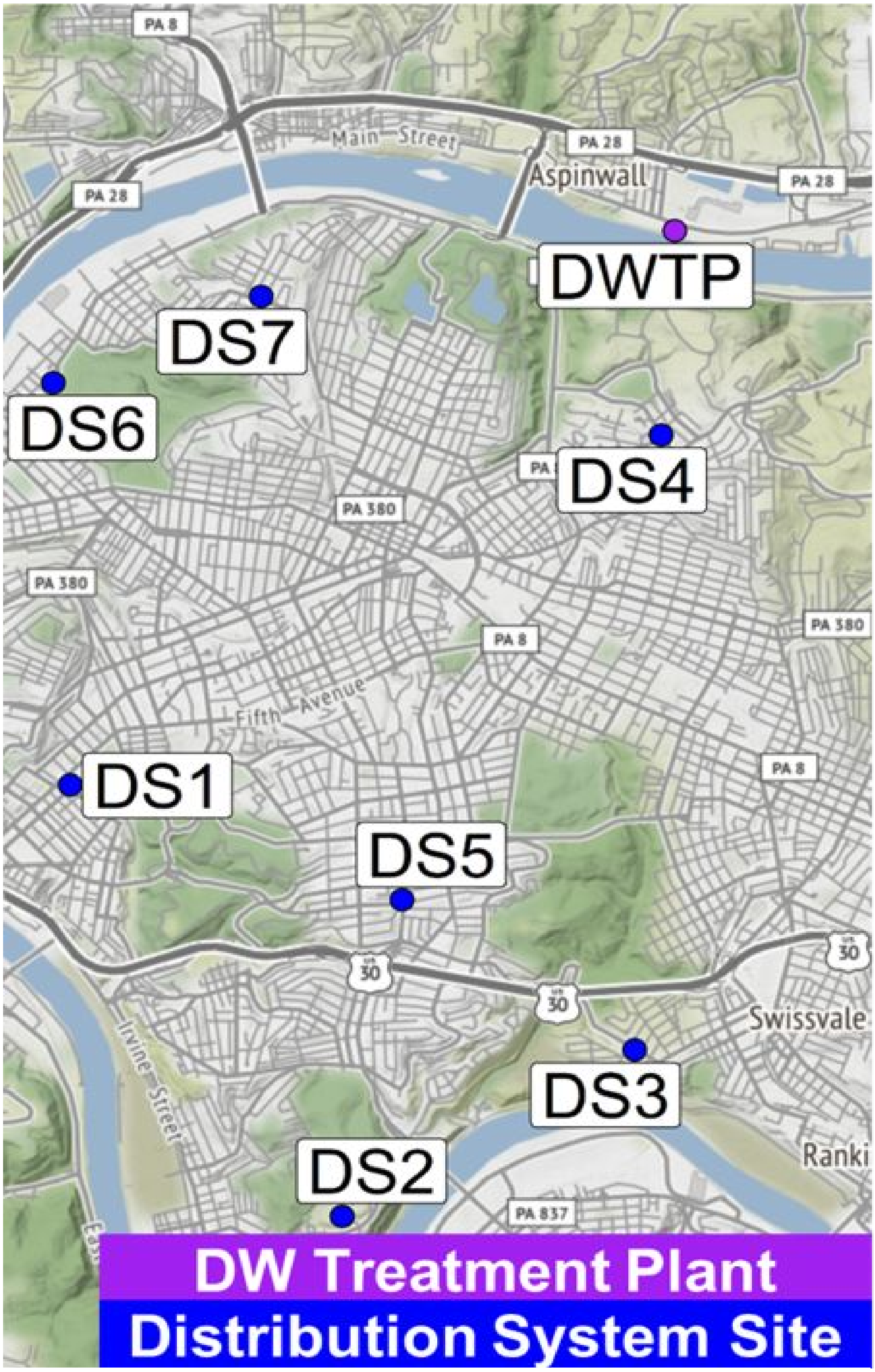

Figure A1: Map of distribution system routine monitoring site sampling locations

Supplementary Information

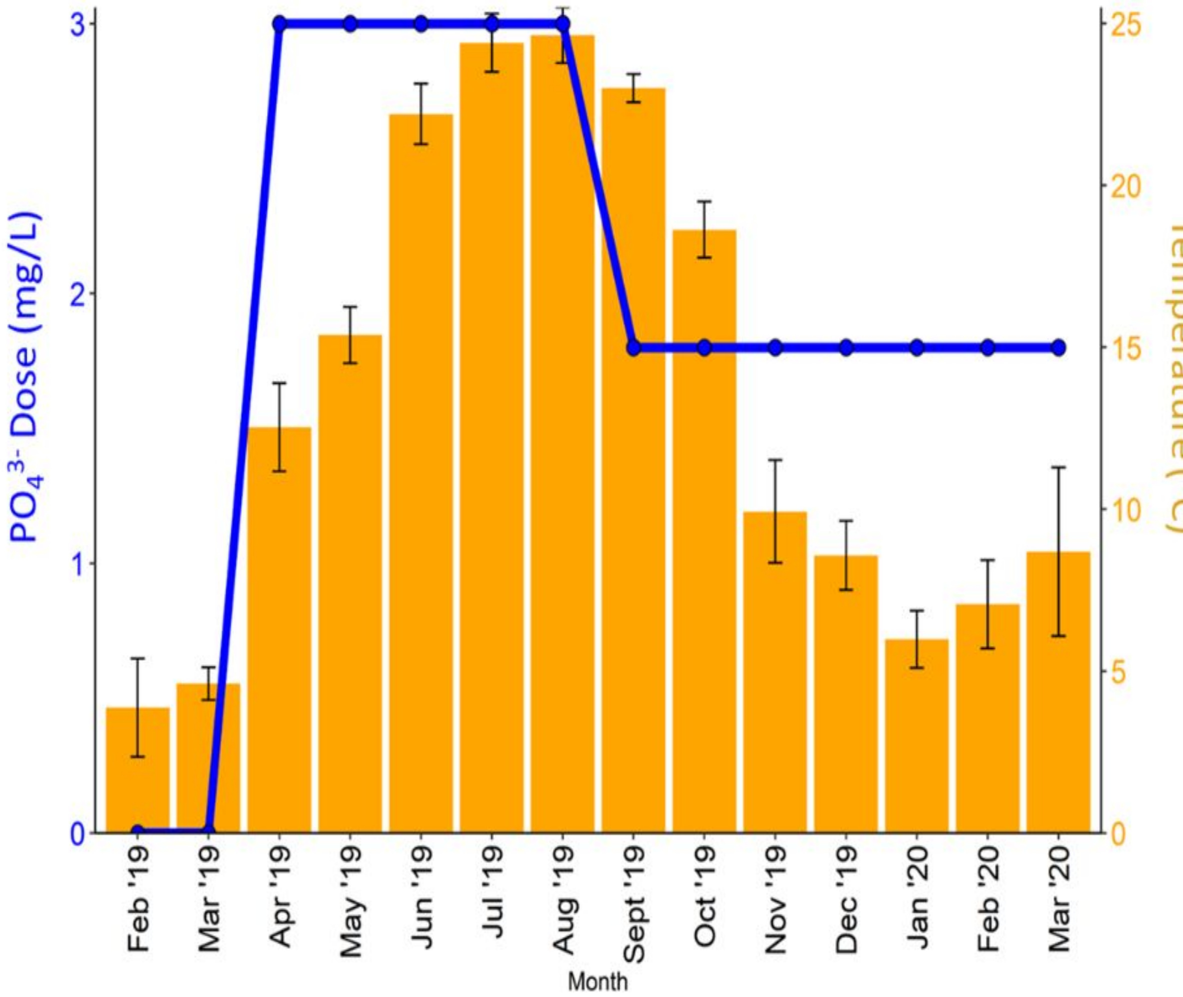

Figure A2: PO_4^{3-} dosing (left scale, in blue) and water temperature (right scale, in orange) over the duration of the one-year study. Water temperature was averaged between the seven distribution sites and error bars represent the standard deviation.

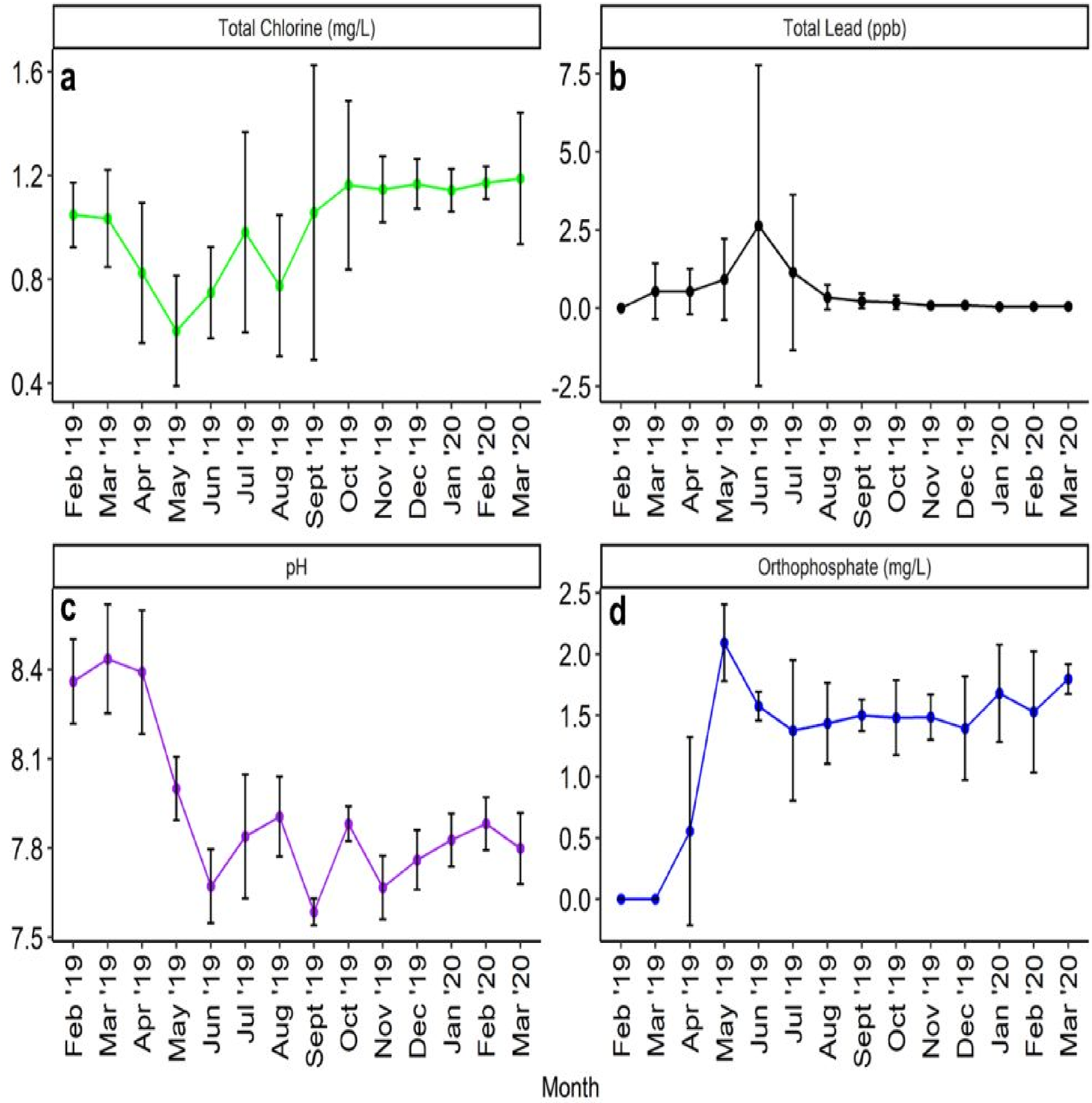

Figure A3: Total chlorine (a), Total lead (b), pH (c), and PO_4^{3-} concentration (d) across the seven distribution system sites over the duration of the one-year study. The PO_4^{3-} concentration measured in the distribution system differs from the dose in Figure A2 due to scale formation.

Supplementary Information

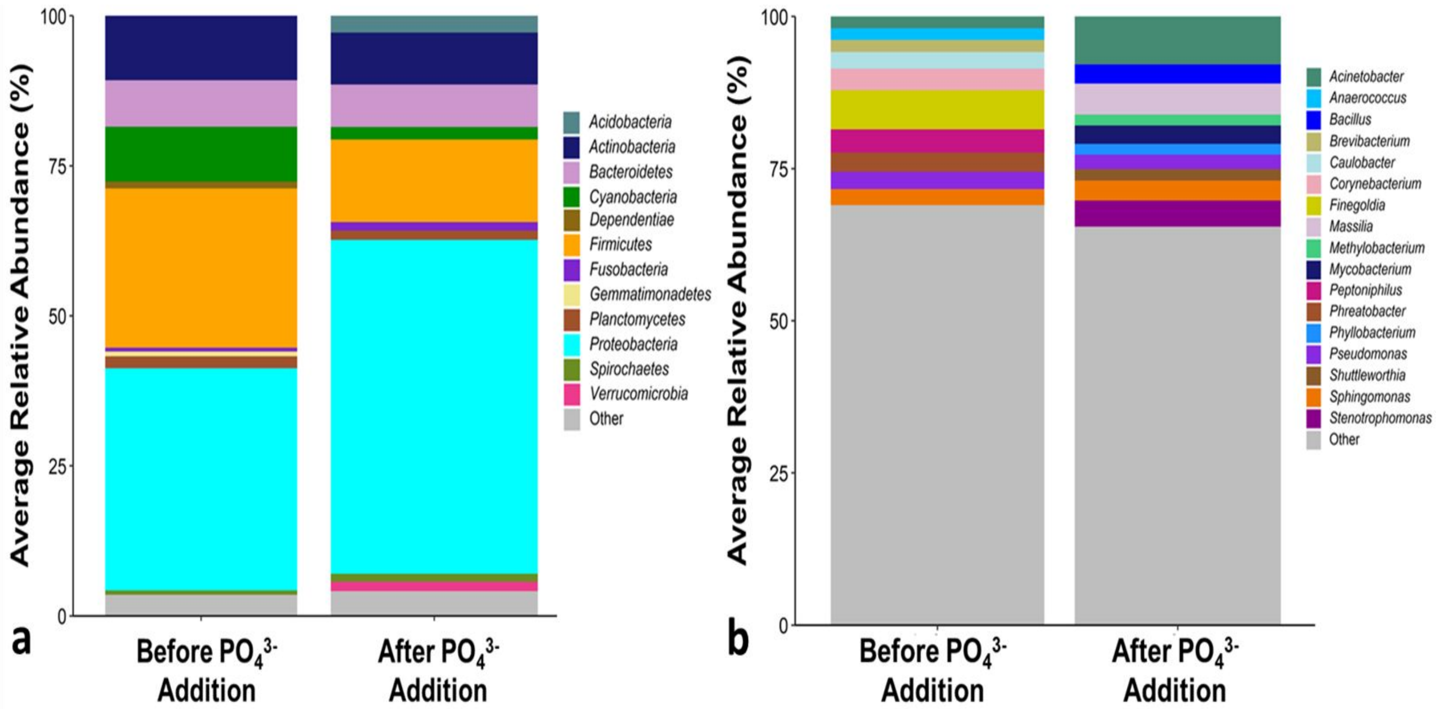

Figure A4: a) Top 10 most abundant phyla and **b)** top 10 most abundant genera across all DWDS sites before and 1-year after full-scale PO_4^{3-} addition (n = 14 in each condition). To control for seasonality, only the matching months before and after PO_4^{3-} application were compared.

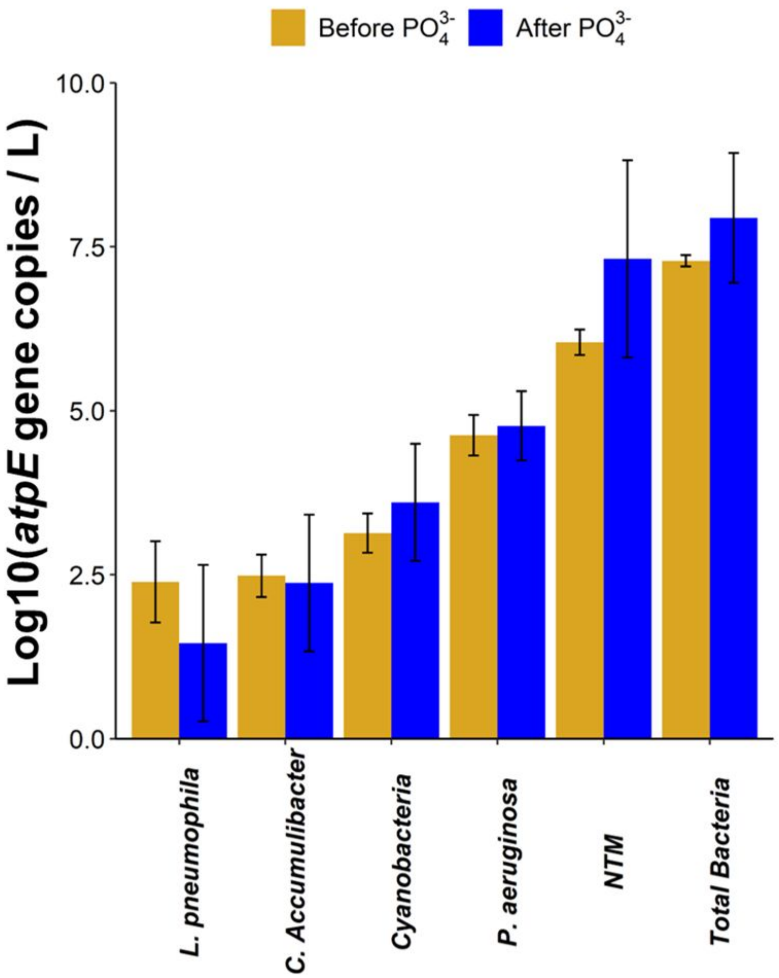

Figure A5: Average ± standard deviation of absolute density of DWPIs, total bacteria, and Cyanobacteria in the DWDS before (n = 21) and after PO_4^{3-} addition (n = 77). The after category contains data from 9 months of sample collection.

Supplementary Information

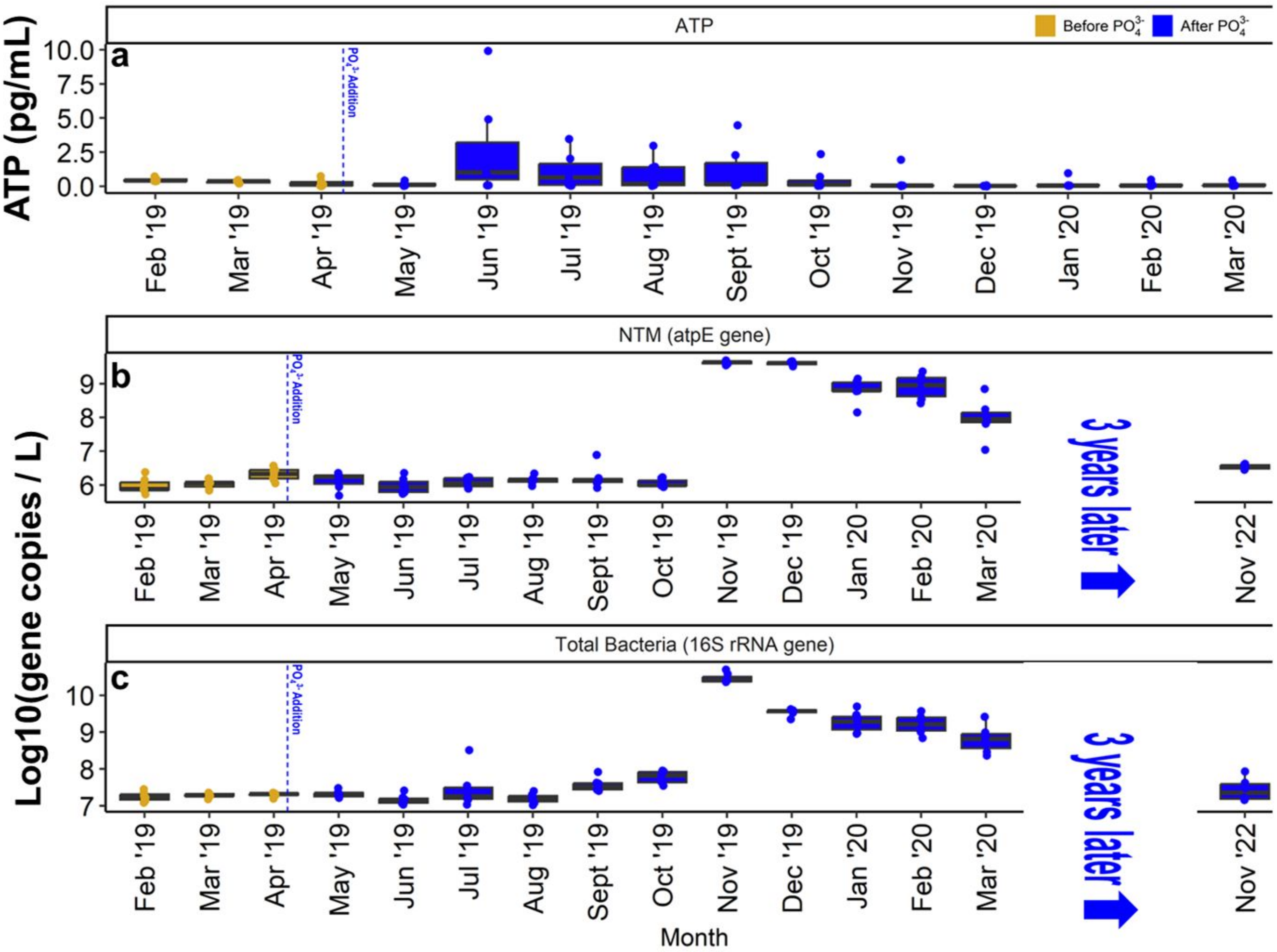

Figure A6: ATP (a), Absolute NTM density (b) and Total bacterial (b) density in the DWDS throughout the study duration and comparison three years later. *Note: only six samples were collected in 2022, as one of the routine monitoring sites has been shut down. No ATP measure was taken in the 2022 samples.*

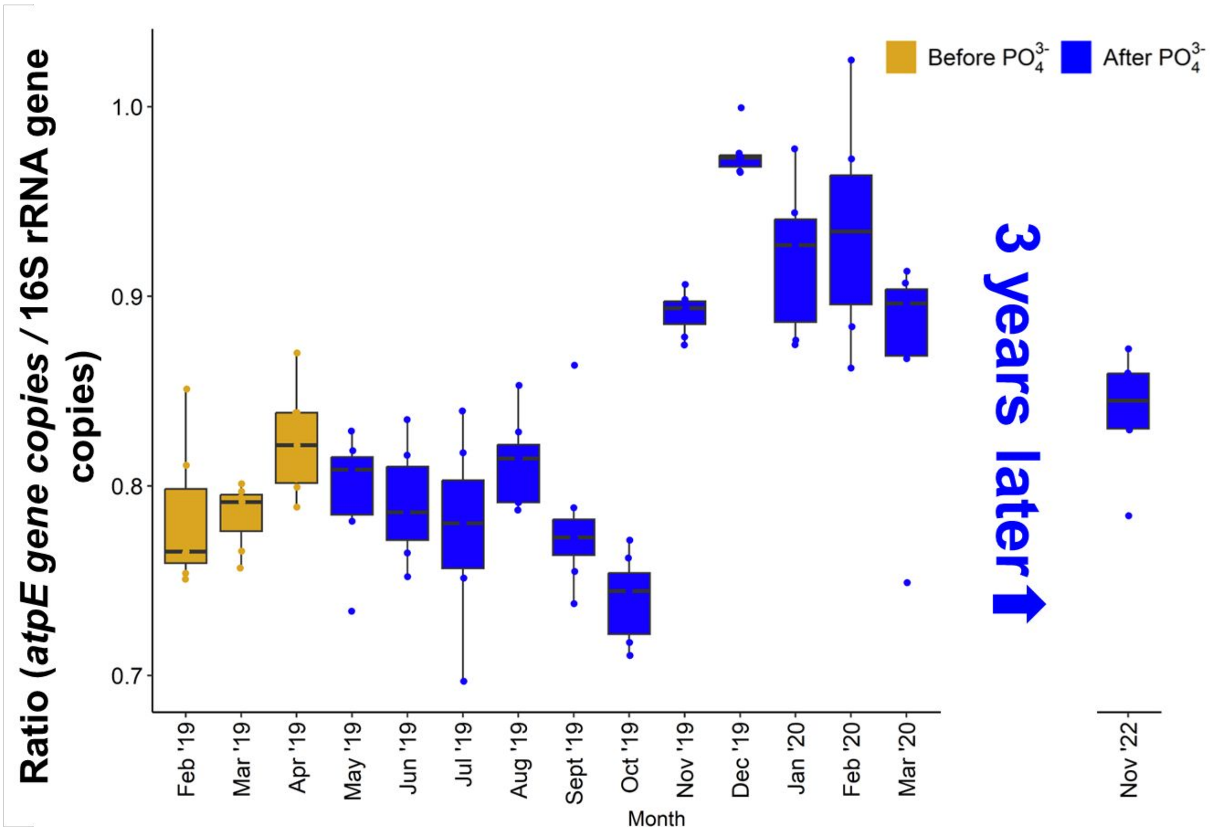

Figure A7: Ratio of NTM (as measured by the *atpE* gene) to total bacteria (as measured by the 16S rRNA gene) across the seven distribution system sites over the course of the study duration and three years later. *Note: only six samples were collected in 2022, as one of the routine monitoring sites has been shut down*

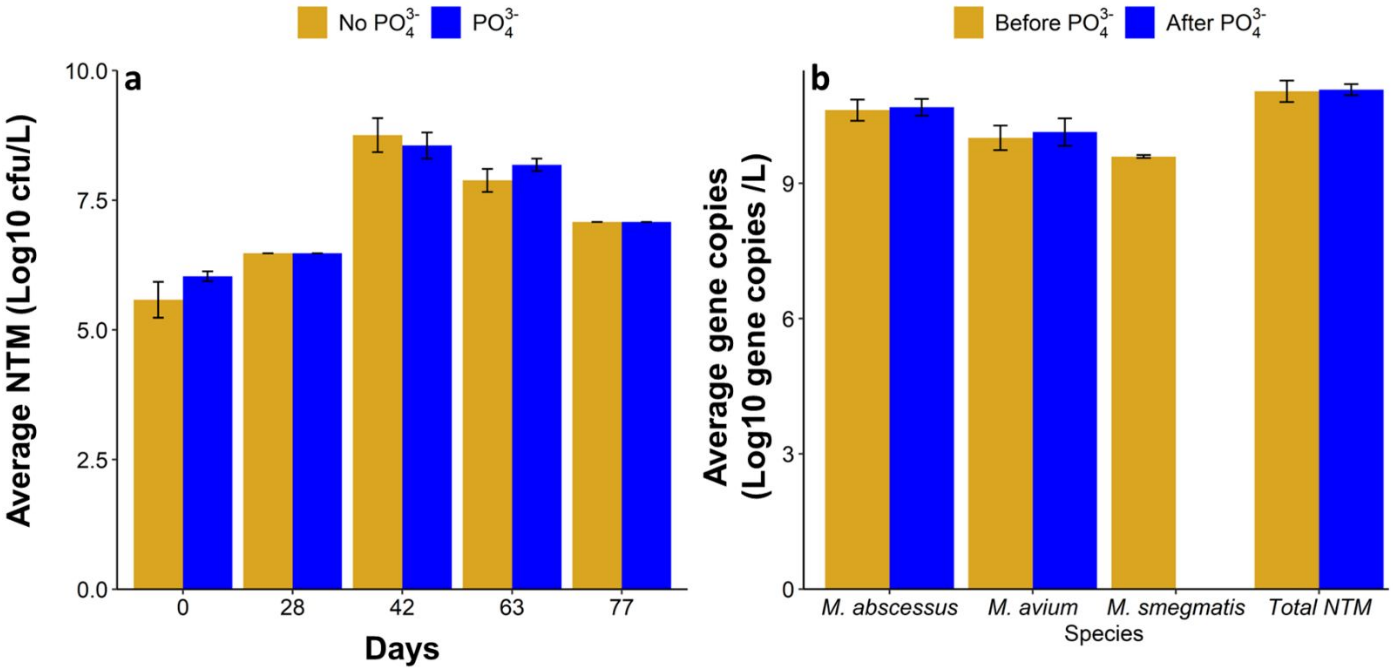

Figure A8: a) NTM concentration over the study duration and **b)** absolute density of NTM species in batch reactors. Over the duration of the experiment (~ 3 months), a significant difference in *M. smegmatis* density was observed, likely due to competition with the environmental *M. avium, M. abcessus,* and other microorganisms present in the water.

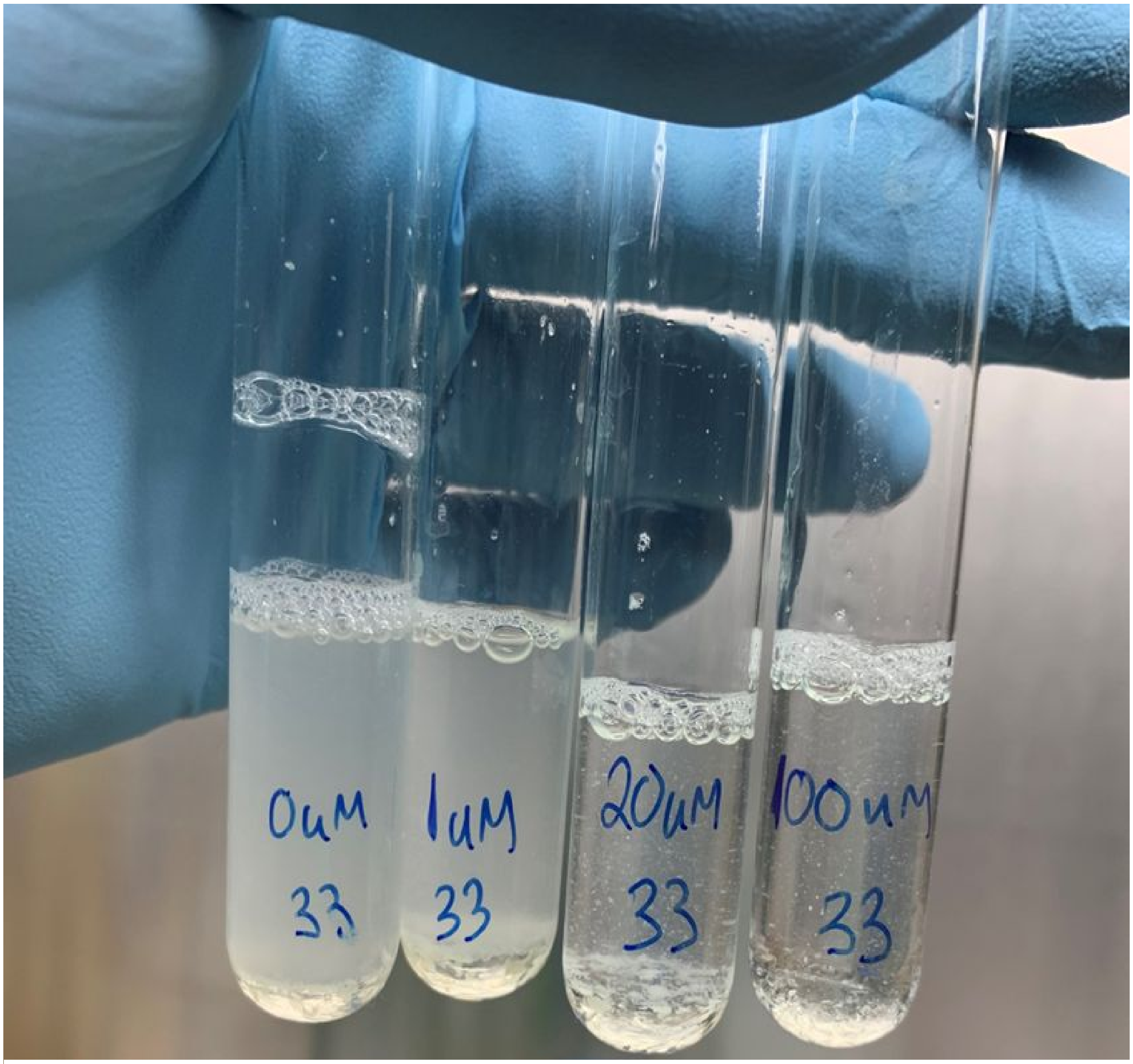

Figure A9: Aggregate fraction of *M. abcessus* cultures suspended in phosphate buffered saline mixed with 6% Tween20. As phosphate level increased, the number of suspended aggregates decreased.

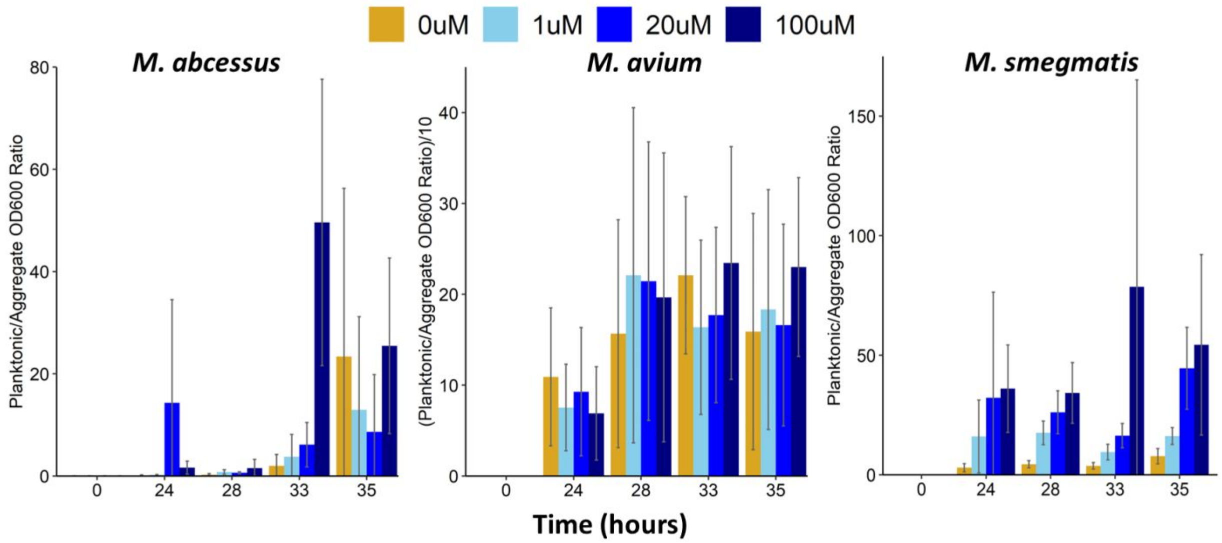

Figure A10: Average (n = *9 for each species, at each timepoint, at each phosphorus concentration*) ± standard deviation of planktonic vs aggregate NTM ratios for *M. abcessus* (left), *M. avium* (middle), and *M. smegmatis* (right). Ratios were obtained by dividing the planktonic OD600 measurements by the aggregate OD600 measurements. A larger ratio signifies a larger amount of NTM in the planktonic phase.

Supplementary Information

Table A1: Different water quality parameters measured in this study and the method / apparatus used

Parameter	Unit	Method / Apparatus
Temperature	℃	Temperature probe
pH	--	pH electrode
Orthophosphate	mg/L PO_4^{3-}	PhosVer3 Ascorbic Acid Method
Total & Dissolved Iron	mg/L	ICPMS (dissolved 0.45 um filtered)
Total & Dissolved Copper	mg/L	ICPMS (dissolved 0.45 um filtered)
Total & Dissolved Manganese	mg/L	ICPMS (dissolved 0.45 um filtered)
Total & Dissolved Lead	µg/L	ICPMS (dissolved 0.45 um filtered)
Total Chlorine	mg/L Cl_2	DPD Method
Free Chlorine	mg/L Cl_2	DPD Method
Turbidity	NTU	Turbidimeter
ATP	mg/L	AquaSnap Total ATP meter

Table A2: Average ± standard deviation (s.d.) measured water quality parameters before and after full-scale PO_4^{3-} addition into the DWDS. Dissolved concentrations are in parentheses.

**Parameter**	**Average ± s.d. Before PO_4^{3-} (n = 14)**	**Average ± s.d. After PO_4^{3-} (n = 14)**
Temperature	4.24 ± 1.19	7.76 ± 2.27
pH	8.40 ± 0.17	7.82 ± 0.11
Orthophosphate	0.001 ± 0.002	1.71 ± 0.364
Total (& Dissolved) Iron	0.028 ± 0.027<br>(0.017 ± 0.017)	0.013 ± 0.016<br>(0.001 ± 0.001)
Total (& Dissolved) Copper	0.005 ± 0.006<br>(0.004 ± 0.005)	0.009 ± 0.004<br>(0.008 ± 0.003)
Total (& Dissolved) Manganese	0.006 ± 0.013<br>(0.001 ± 0.002)	0.001 ± 0.001<br>(0.0001 ± 0.0001)
Total (& Dissolved) Lead	0.3 ± 1.0<br>(0.057 ± 0.062)	0.051 ± 0.043<br>(0.01 ± 0.01)
Total Chlorine	1.04 ± 0.16	1.17 ± 0.19
Free Chlorine	0.94 ± 0.16	1.06 ± 0.18
Turbidity	0.068 ± 0.031	0.102 ± 0.065
ATP	$4.1 \times 10^{-7} \pm 1.3 \times 10^{-7}$	$1.2 \times 10^{-7} \pm 1.4 \times 10^{-7}$

Supplementary Information

Table A3: ddPCR assay thresholds, LOD, and LOQ

Target taxa (gene)	ddPCR Threshold	Limit of Detection	Limit of Quantification
Total Bacteria	12900	5.3 gene copies / 20 µL	53 gene copies / 20 µL
L. pneumophila	8800	6.08 gene copies / 20 µL	6.08 gene copies / 20 µL
P. aeruginosa	4500	7.3 gene copies / 20 µL	7.3 gene copies / 20 µL
NTM	10600	5.6 gene copies / 20 µL	5.6 gene copies / 20 µL
M. abcessus	9093	6.62 gene copies / 20 µL	6.62 gene copies / 20 µL
M. avium	4391	6.62 gene copies / 20 µL	66 gene copies / 20 µL
M. smegmatis	11643	5.8 gene copies / 20 µL	5.8 gene copies / 20 µL
Cyanobacteria	9567	7.9 gene copies / 20 µL	7.9 gene copies / 20 µL

Thresholds were determined experimentally by spiking target taxa's gblock at different concentrations into water DNA matrix and adjusting threshold until expected concentration was read out.

Table A4: ddPCR target genes, amplicon size, annealing temperature, and primer sequences

Target Taxa	Target gene	Approx. Amplicon Size (bp)	Annealing Temp. (℃)	Sequence (5' to 3')
Total Bacteria	16S rRNA	200	57	F: ACTCCTACGGGAGGCAG
				R: ATTACCGCGGCTGCTGG
Legionella pneumophila	*Lmip*	150	57	F: CCGATGCCACATCATTAGC
				R: CCAATTGAGCGCCACTCATAG
Pseudomonas aeruginosa	*Orpl*	117	57	F: CGAGTACAACATGGCTCTGG
				R: ACCGGACGCTCTTTACCATA
Nontuberculous mycobacteria	*atpE*	164	57	F: CGGYGCCGGTATCGGYGA
				R: CGAAGACGAACARSGCCAT
Mycobacterium abcessus	*rpoB*	77	56.5	F: CGATAGAGGACTTCGCCTAACC
				R: TCGAGCACGTAAACTCCCTTC
Mycobacterium avium	16S rRNA	97	55.5	F: GGGTGAGTAACACGTGTGCAA
				R: CCAGAAGACATGCGTCGTGA
Mycobacterium smegmatis	*rrnB*	75	60.7	F: ATCCTCGCTGCCACTAGAGA
				R: AAACAACACGCCCGACTTTG
Cyanobacteria	16S rRNA	422	60	F: GGGGAATCTTCCGCAATGGG
				R: GACTACTGGGGTATCTAATCCCATT

Supplementary Information

Table A5: ddPCR reaction conditions

Target taxa (gene)	Temperatures and Times	# of cycles
Total Bacteria, *L. pneumophila, P. aeruginosa,* NTM	95°C, 5:00, Ramp 2/s<br>95°C, 0:30, Ramp 2/s<br>57°C, 1:00, Ramp 2/s<br>72°C, 1:00, Ramp 2/s<br>4°C, 5:00, Ramp 2/s<br>90°C, 5:00, Ramp 2/s<br>12°C, --, Ramp 2/s	45
M. abcessus	95°C, 5:00, Ramp 2/s<br>95°C, 0:30, Ramp 2/s<br>56.5°C, 1:00, Ramp 2/s<br>72°C, 1:00, Ramp 2/s<br>4°C, 5:00, Ramp 2/s<br>90°C, 5:00, Ramp 2/s<br>12°C, --, Ramp 2/s	45
M. avium	95°C, 5:00, Ramp 2/s<br>95°C, 0:30, Ramp 2/s<br>55.5°C, 1:00, Ramp 2/s<br>72°C, 1:00, Ramp 2/s<br>4°C, 5:00, Ramp 2/s<br>90°C, 5:00, Ramp 2/s<br>12°C, --, Ramp 2/s	45
M. smegmatis	95°C, 5:00, Ramp 2/s<br>95°C, 0:30, Ramp 2/s<br>60.7°C, 1:00, Ramp 2/s<br>72°C, 1:00, Ramp 2/s<br>4°C, 5:00, Ramp 2/s<br>90°C, 5:00, Ramp 2/s<br>12°C, --, Ramp 2/s	45
Cyanobacteria	95°C, 5:00, Ramp 2/s<br>95°C, 0:30, Ramp 2/s<br>60°C, 1:00, Ramp 2/s<br>72°C, 1:00, Ramp 2/s<br>4°C, 5:00, Ramp 2/s<br>90°C, 5:00, Ramp 2/s<br>12°C, --, Ramp 2/s	44
